# Neuroprotective Effects of Oligosaccharides in Rehmanniae Radix on Transgenic *Caenorhabditis elegans* Models for Alzheimer’s Disease

**DOI:** 10.3389/fphar.2022.878631

**Published:** 2022-06-17

**Authors:** Nianxin Kang, Yage Luan, Yu Jiang, Wenhao Cheng, Yongjian Liu, Zhijun Su, Yonggang Liu, Peng Tan

**Affiliations:** Beijing Key Laboratory for Production Process Control and Quality Evaluation of Traditional Chinese Medicine, Beijing Municipal Science and Technology Commission, School of Chinese Pharmacy, Beijing University of Chinese Medicine, Beijing, China

**Keywords:** Rehmanniae radix, oligosaccharide, *Caenorhabditis elegans*, Alzheimer’s disease, lifespan, amyloid-beta deposition, antioxidant

## Abstract

Rehmanniae Radix (RR, the dried tuberous roots of *Rehmannia glutinosa* (Gaertn.) DC.) is an important traditional Chinese medicine distributed in Henan, Hebei, Inner Mongolia, and Northeast in China. RR is frequently used to treat diabetes mellitus, cardiovascular disease, osteoporosis and aging-related diseases in a class of prescriptions. The oligosaccharides and catalpol in RR have been confirmed to have neuroprotective effects. However, there are few studies on the anti-Alzheimer’s disease (AD) effect of oligosaccharides in Rehmanniae Radix (ORR). The chemical components and pharmacological effects of dried Rehmannia Radix (DRR) and prepared Rehmannia Radix (PRR) are different because of the different processing methods. ORR has neuroprotective potential, such as improving learning and memory in rats. Therefore, this study aimed to prove the importance of oligosaccharides in DRR (ODRR) and PRR (OPRR) for AD based on the *Caenorhabditis elegans* (*C. elegans*) model and the different roles of ODRR and OPRR in the treatment of AD. In this study, we used paralysis assays, lifespan and stress resistance assays, bacterial growth curve, developmental and behavioral parameters, and ability of learning and memory to explore the effects of ODRR and OPRR on anti-AD and anti-aging. Furthermore, the accumulation of reactive oxygen species (ROS); deposition of Aβ; and expression of amy-1, sir-2.1, daf-16, sod-3, skn-1, and hsp-16.2 were analyzed to confirm the efficacy of ODRR and OPRR. OPRR was more effective than ODRR in delaying the paralysis, improving learning ability, and prolonging the lifespan of *C. elegans*. Further mechanism studies showed that the accumulation of ROS, aggregation, and toxicity of Aβ were reduced, suggesting that ORR alleviated Aβ-induced toxicity, in part, through antioxidant activity and Aβ aggregation inhibiting. The expression of amy-1 was downregulated, and sir-2.1, daf-16, sod-3, and hsp-16.2 were upregulated. Thus, ORR could have a possible therapeutic effect on AD by modulating the expression of amy-1, sir-2.1, daf-16, sod-3, and hsp-16.2. Furthermore, ORR promoted the nuclear localization of daf-16 and further increased the expression of sod-3 and hsp-16.2, which significantly contributed to inhibiting the Aβ toxicity and enhancing oxidative stress resistance. In summary, the study provided a new idea for the development of ORR.

## 1 Introduction

Alzheimer’s disease (AD), which dates back to 1907 (O'Brien and Wong, 2011), lowly and irreversibly destroys cognition, memory, and eventually the ability to carry out daily activities, leading to the need for full-time care. The most recent data indicated that, by 2050, the prevalence of dementia will double in Europe and triple worldwide (Scheltens et al., 2021). What is required for the pathological diagnosis of AD is the spread of amyloid-beta (Aβ) and tau. The pathological diagnosis is the gold standard for identifying AD (Sengoku, 2020). In healthy neurons, the amyloid precursor protein (APP) has three domains (intracellular, intercellular, and extracellular), which will be digested by α- and γ-secretase enzymes. However, when β- and γ-secretsase cooperate, this digestive reaction produces Aβ, an insoluble peptide. Amyloid peptides clump together and form beta-amyloid plaques (ABP), which are deteriorative for the cells (Ashrafian et al., 2021). ABP can damage the brain and make it lose some functions, such as memory. It may also damage peripheral neurons by causing inflammation. Based on this, several natural products have been proposed as the potential inhibitors of Aβ formation, such as resveratrol, major compound from the Sappan wood brazilin, and Ligustilide from Ligusticum chuanxiong (Guo et al., 2017; Xu et al., 2018; Al-Edresi et al., 2020).

Neurodegenerative diseases, including AD, represent a significant and growing burden around the world. The verification of factors that decrease their impact requires a plethora of research efforts. Considerable scientific research has contributed significantly to the mechanism involved in the disease progression of AD and the discovery of treatment using biomodels, such as rats, mice, cell lines, and *Caenorhabditis elegans* (*C. elegans*) (Shanmuganathan et al., 2019; Zhu et al., 2019). Currently, the mammalian as an *in vivo* model for the selection of anti-AD drug candidates has some limitations, including the cost and time of the experiment and the number and quality of the study subjects (Alexander et al., 2014). In addition, mammalian neuronal cell culture *in vitro* cannot completely simulate the complexities of the human brain (Paul et al., 2020). Therefore, it is necessary to explore alternative experimental models for screening anti-AD drugs. *C. elegans* is a harmless, free-living nematode that feeds on microorganisms. Although the lack of evolutionary complexity, many of the cellular mechanisms and molecular pathways of *C. elegans* are conserved with those of mammals, therefore allowing it for comparative studies. 41% of the *C. elegans* genes have human orthologs, and about 83% of *C. elegans* proteome have human homolog genes (Lai et al., 2000; Kim et al., 2018)*.* Moreover, the *C. elegans* genome possesses homologs of around two-thirds of all human disease genes. Previous research has confirmed that 108 genes were human orthologs by performing a functional analysis of 143 essential genes, in which 97 genes were related to 1,218 different diseases (Qin et al., 2018). *C. elegans* has become a very popular model organism suited for high throughput studies due to its small size, fast generation cycle, high fecundity, cheap culturing conditions, and complete genome sequencing. In recent years, scientists have constructed a variety of transgenic nematodes to screen AD drug candidates and establish the molecular dynamics of proteins and genes (β-sheet breaking property, Aβ expression level, hyperphosphorylation of Tau, and many more) associated with AD (Morales-Zavala et al., 2017; Ahmad, 2018; Sarasija et al., 2018; Zhu et al., 2019). It is well known that the expression pattern of Aβ peptide is an important parameter in the study of AD pathology in miniaturized models such as *C. elegans*. However, a study says that Apl-1, an APP family protein present in *C. elegans,* will not yield an Aβ_42_ peptide due to the absence of β-secretase (Priller et al., 2006). This ensures the compatibility of the transgenic model of *C. elegans* such as CL4176, GMC101, CL2006, and CL2355 to study the possible expression patterns of the Aβ_42_ peptide. The researchers have indicated the potential of herbal drugs to treat AD using these models. A study indicated the efficacy of Norcrassin A, a novel C16 tetranorditerpenoid obtained from the roots of *Croton crassifolius* by suppressing Aβ induced paralysis (Zhang Z. X. et al., 2018). *Hibiscus sabdariffa* L. extract resulted in a prominent extension of lifespan and protected against Aβ toxicity in the model organism of *C. elegans* (Koch et al., 2020). Delayed Aβ-induced paralyses were observed in CL4176 transgenic worms treated with *Betula utilis* ethanolic extract (Pandey et al., 2020). Furthermore, the reports have screened abundant therapeutic agents such as *Coptis chinensis* Franch. polysaccharide, Guarana (*Paullinia cupana* Mart.) ethanolic extract, *Padina gymnospora*, and its active constituent against various study parameters using *C. elegans* (Li et al., 2018; Shanmuganathan et al., 2019; Zamberlan et al., 2020).

Rehmanniae Radix (RR), the dried tuberous roots of *Rehmannia glutinosa* (Gaertn.) DC., is a traditional Chinese medicine recorded in Shen Nong’s Herbal Classics (National Pharmacopoeia Commission., 2020), which is mainly produced in Henan, Hebei, Inner Mongolia, and Northeast in China. According to different processing methods, dried Rehmannia Radix (DRR) and prepared Rehmannia Radix (PRR) are used in a large number of traditional Chinese medicine prescriptions with completely different effects, including diabetes mellitus, cardiovascular disease, osteoporosis (Zhang et al., 2008.), chemotherapy-induced adverse effects prevention (Shi et al., 2016) and neurodegenerative disorder (Sanabria, 2013). DRR is good at clearing heat and cooling blood, while PRR plays a role in tonifying blood and nourishing Yin (Yuan et al., 2019). PRR is commonly used for the treatment of aging-related diseases due to its nourishing property. Liuwei Dihuang (LWDH), a traditional Chinese medicinal formula containing PRR, could alleviate the paralytic phenotype of the *C. elegans* model of Alzheimer’s disease (Zhang Y. et al., 2018). The difference in their efficacy is related to the change in composition caused by processing. Liang et al. (2013) confirmed significant differences in the composition of fructose, rehmaionoside, and stachyose in DRR and PRR, which may cause their different efficacy. Catalpol, an iridoid glycoside component, is also decomposed through steaming and is not suitable as a characteristic component of PRR.

Oligosaccharides from medicinal plants have increasingly attracted widespread attention at home and abroad because of their significant biological activities and great medicinal potential. Many studies showed that oligosaccharides possess various activities, such as antioxidation, neuroprotection, anti-infection, and intestinal microbiota regulation (Chen et al., 2015; Liu et al., 2021). GV-971, a mixture of acidic linear oligosaccharides extracted from marine brown algae, could cross the blood–brain barrier to directly bind to Aβ and inhibit the fibril formation of Aβ. In November 2019, it was approved by China’s regulators to treat mild-to-moderate AD (Zhang et al., 2021). Prebiotic oligosaccharides, including manno-oligosaccharides (MO), galacto-oligosaccharides (GOS), fructo-oligosaccharides (FOS), xylo-oligosaccharides (XOS), have exhibited the potential to increase memory, cognition ability, and social behavior. Their health attributes have been widely reviewed and are accepted globally (Divyashri et al., 2021). As chemical components with high content in RR, polysaccharides and oligosaccharides vary greatly during processing (Zhou et al., 2016). Oligosaccharides in RR could reduce blood glucose (Zhang et al., 2014) and provide neuroprotection, such as improving learning and memory ability in rats with dementia and diabetes (Yang et al., 2008) and ameliorating hippocampal tissue damage (Blasco et al., 2014).

In this study, we analyzed the potential of oligosaccharides of dried Rehmannia Radix (ODRR) and prepared Rehmannia Radix (OPRR) in the treatment of AD from the aspects of motor phenotype, learning and memory ability, anti-aging, growth, and development. In addition, experiments such as oxidative stress, determination of ROS, Aβ protein staining, and gene expression analysis were also performed to determine the mechanism of ORR.

## 2 Materials and Methods

### 2.1 *C. elegans* Strains and Handling Conditions

The wild-type strains N2, CL4176 [*smg-1*(*myo-3/Aβ*_*1-42*_ */letUTR*)*+pRF4*], CL2006 [*pCL12*(*unc-54/Aβ*_*1-42*_)*+pRF4*], CL2355 [*pCL45*(*snb-1::Aβ*_*1-42*_ *::3′UTR*(*long*)*+mtl-2::GFP*], and its control strain CL2122 [*unc-54vector + mtl-2::GFP*], CF1553 [(*pAD76*)*sod-3::GFP + rol6(su1006)*], LD1 (ldIs7 [skn-1b/c::GFP + rol-6 (su1006)]), TJ356 (zIs356 [daf-16p::daf-16a/b::GFP + rol-6 (su1006)]), and TJ375 (gpIs1 [hsp-16.2::GFP]) were used in this study, which was obtained from Caenorhabditis Genetics Center (CGC, University of Minnesota, MN, United States). All strains were cultivated on nematode growth medium (NGM) plates seeded with living *E. coli* OP50 as food and incubated at 20°C, except for strains CL4176, CL2355, and CL2122, which were maintained at 16°C. For all worms, age-synchronized eggs were obtained using bleach (0.5 M NaOH, 5% NaClO) to separate embryos from the pregnant hermaphrodite. Strain *E. coli* OP50 was grown in LB at 37°C for 24 h in an incubator.

### 2.2 Oligosaccharide Preparation From Dried Rehmanniae Radix and Prepared Rehmanniae Radix

Dried Rehmannia Radix was provided by Beijing Shuangqiao Yanjing Chinese Medicine Decoction Pieces Factory (Beijing, China). The materials were carefully authenticated by one of the authors, Prof. Peng Tan. DRR was steamed to get PRR. Their powders were refluxed with 10 times of water for three times, and the extracts were combined and concentrated to 1 mg/ml. 95% ethanol was added to the concentrate until the ethanol content was 80% and centrifuged at 10,000 rpm for 15 min to remove polysaccharides and proteins. The supernatant was concentrated and freeze-dried to prepare crude oligosaccharides. Finally, the crude oligosaccharides were purified with Sephadex LH-20 to obtain a partially purified oligosaccharide-enriched fraction.

### 2.3 Characterization of Chemical Profile of ORR

We carried out the content determination method analysis of ODRR and OPRR and modified the previous method (Chiu et al., 2018). The analysis was performed on a SeQuant^®^ZIC^®^-HILIC (Merck Millipore, Darmstadt, Germany). The evaporative light scattering detector (ELSD) was used with a drift tube temperature of 50°C. The mobile phase was A: 0.5 mM ammonium acetate aqueous solution (containing 0.1% formic acid), B: acetonitrile containing 0.1% formic acid, with a program of 80-20%B in 0–19 min, 20-80%B in 19–25 min, and 80%B in 25–30 min. The injection volume was 20 μL with a mobile phase flow rate of 1.0 ml/min.

### 2.4 Paralysis Assays

In order to determine whether ORR inhibits or delays the onset of progressive paralysis induced by Aβ in CL4176 expressing the Aβ_1-42_ peptide in muscle tissue, synchronized L1 stage CL4176 worms were transferred to NGM plates containing different concentrations of 1, 2, or 5 mg/ml ODRR and OPRR, respectively. The worms were shifted to 23°C after incubating for 48 h at 16°C to initiate the Aβ-induced paralysis. Scoring began at 2 h intervals after 36 h at 23°C. The nematodes were assessed as paralyzed on the basis of either immobility with a touch of a platinum loop on the NGM or the presence of clustered eggs in the worm’s abdomen. Each experiment was performed with about 50 worms per group three times.

### 2.5 Lifespan and Stress Resistance Assays

CL4176 nematodes synchronized at the L4 stage were exposed to 2 mg/ml ODRR and OPRR or OP50, respectively, until adulthood. Survival analysis was implemented to count the number of nematodes surviving every day from the egg stage at 16°C to the death of all worms. Each group has about 90 animals divided into three NGM plates.

Oxidative stress was assessed in CL4176 treated with 2 mg/ml ODRR and OPRR or OP50 from L1 stage to adults. The adults were then transferred to NGM containing 300 μM juglone, and the number of living and dead nematodes was counted every hour. Three independent experiments were performed, each with 30 nematodes.

### 2.6 Analysis of Developmental and Behavioral Parameters

To assay, synchronized L4 stage CL4176 worms were transferred to NGM plates, and aliquots of 2 mg/ml ODRR or OPRR were placed onto plates. The adult nematodes were transferred to freshly prepared NGM plates every day until the end of the reproductive period. The hatched offspring were counted 2 days later. The number of nematodes used for the experiment was 12.

Thrash frequency was selected for locomotion analysis. Synchronized L1 larvae of N2 nematodes were incubated with 2 mg/ml ODRR and OPRR at 20°C until adulthood. These worms were collected and placed on glass slides dripped with S-basal buffer on Day 4 and Day 8 after adulthood and then allowed to stand for 30 s. Then, the number of body bendings of the individual nematode was counted under a microscope for 20 s. Body bending was defined as a change in the direction of mid-body bending. Twenty nematodes were counted approximately in each treatment.

For the measurement of pharyngeal pumping rates, synchronized N2 was treated with 2 mg/ml ODRR or OPRR for 48 h at 20°C from L1 to 1 day of adulthood. Then, the worms were transferred to fresh NGM plates, and pumping rates were analyzed in 20 s for every worm. Each experiment was performed with about 20 worms per group three times.

### 2.7 Bacterial Growth Curve

The effect of ORR on the growth of *E. coli* OP50 in 5 days was determined. *E. coli* OP50 was inoculated into an LB medium and incubated at 37°C for 24 h. Next, ODRR and OPRR were added respectively to reach the concentration of 2 mg/ml and incubated at 37°C for 5 days. The OD_600_ of OP50 in each group was determined every 24 h.

### 2.8 Measurement of Intracellular Reactive Oxygen Species

Intracellular ROS in CL4176 strains was measured using H_2_DCF-DA. The synchronized CL4176 nematodes exposed to ODRR and OPRR were incubated at 16°C for 48 h and then transferred to 23°C. The nematodes were collected and washed twice with S-basal buffer after the temperature was raised for 36 h. Subsequently, 20 μM H_2_DCF-DA was added to a centrifuge tube containing 30 nematodes and incubated at 37°C in dark for 30 min. Nematodes were washed with S-basal and transferred to the agar pad. Fluorescence was measured in triplicate using a fluorescence microscope with excitation wavelengths at 485 nm and emission at 535 nm. Fluorescence intensity was quantified by ImageJ software. Approximately 20 worms per group were measured in each analysis.

### 2.9 Aβ Analysis With ThS

The transgenic nematodes CL2006 expressing high level of human Aβ in body wall cells were cultured on NGM with or without ODRR or OPRR at 20°C. The worms were collected and washed twice with S-basal and transferred to centrifuge tubes at the fifth day of the age. Nematodes were fixed in phosphate-buffered saline (PBS) containing 4% paraformaldehyde at 4°C for 24 h. Then, they were infiltrated with Tris (125 mM, pH 7.4) containing 5% β-mercaptoethanol and 1% Triton x-100 at 37°C for 24 h. Next, the worms were stained with 0.125% Thioflavin-S (ThS) in 50% ethanol for 2 min. Then, the samples were washed and decolorized with 50%, 75%, 90%, 75%, and 50% ethanol in turn and transferred to a glass slide for preparation. The number of amyloid plaques on the head of nematodes was scored under a fluorescence microscope. Data are expressed in terms of Aβ deposition/head area. About 15 nematodes were detected in each group three parallel times.

### 2.10 Chemotaxis Assays

Synchronized transgenic *C. elegans* CL2355, expressing Aβ protein in pan-neurons, was incubated with or without ORR starting from the eggs, and CL2122 was the control strain. They were cultured at 16°C for 48 h and then cultured at 23°C for 36 h to induce Aβ expression. Subsequently, the nematodes were placed in the center of the 60 mm NGM plates divided into two quadrants, in which 1 μL 100% ethanol and 0.1% benzaldehyde in ethanol were dropped 1.5 cm from the center, respectively (Figure 6A). 1 µl of 0.25 M sodium azide was also dropped both in the “attractant” spot and on the opposite side. The assay plates were incubated at 23°C for 1 h, and then the number of worms closer to two points was counted. The Chemotaxis index (CI) was calculated with formula CI = (number of nematodes in benzaldehyde position-number of nematodes in ethanol position)/total number of scored nematodes. Nematodes incapable of moving or crawling off the surface of NGM were censored. In each analysis, about 50 animals were measured in each group.

### 2.11 Serotonin Assay

In order to measure the level of Aβ-induced serotonin (5-HT) hypersensitivity, synchronized CL2355 nematodes were treated at 16°C for 48 h with or without ODRR or OPRR and then cultured at 23°C to promote the expression of Aβ protein in neurons, with CL2122 as the control strain. The worms were collected after 36 h with the temperature rising and then transferred to a 96-well plate containing 5 mg/ml 5-HT. The nematodes were assessed as active or paralyzed after 5 min. About 30 nematodes were detected in each group three parallel times.

### 2.12 Quantitative Real-Time Polymerase Chain Reaction (RT-PCR)

The transcripts of amy-1, daf-16, sod-3, hsp-16.2, sir-2.1, and skn-1 were tested to correlate the phenotype and biochemical response of ORR-treated strains with the corresponding genes. The transgenic *C. elegans* strain CL4176 was treated with ODRR or OPRR as described in the earlier section. First of all, the tissue was put into a pre-cooled mortar and ground. RNA was extracted with total RNA extraction reagent TRNzol according to the instructions after the samples were powdered. The total RNA samples were then processed to remove genomic DNA contamination and reverse-transcribe the cDNA using the PrimeScript™ RT Reagent Kit with GDNA Eraser. Real-time quantitative PCR was performed using gene-specific primers (Table 1). The expression of the GAPDH gene was used as the internal control. The reaction procedure was first carried out in 40 cycles (95°C, 5 s; 60°C, 40 s), at 95°C for 30 s, extended after that, and then 10 s at 95°C, 60 s at 60°C, and 15 s at 95°C to establish the melting curve of the PCR product, finally heating from 60°C to 99°C slowly. Data were analyzed with the 2^−△△CT^ method from three independent runs and expressed as mean ± SE.

**TABLE 1:** Gene-specific primers used in RT-PCR.

Genes	Primers
amy-1	Forward, 5′-CAG​AAT​TCC​GAC​ATG​ACT​CAG​GAT​ATG​AAG-3′
	Reverse, 5′-CCC​ACC​ATG​AGT​CCA​ATG​ATT​GC-3′
sir-2.1	Forward, 5′-AGA​ACG​CGC​ATT​TCG​CCA​TAT​TAA​G-3′
	Reverse, 5′-ATA​CTG​ACA​CTC​CAG​CGC​CAG-3′
daf-16	Forward, 5′-TTT​CCG​TCC​CCG​AAC​TCA​A-3′
	Reverse, 5′-ATT​CGC​CAA​CCC​ATG​ATG​G-3′
sod-3	Forward, 5′-AGC​ATC​ATG​CCA​CCT​ACG​TGA-3′
	Reverse, 5′-CAC​CAC​CAT​TGA​ATT​TCA​GCG-3′
hsp16.2	Forward, 5′-ACG​CCA​ATT​TGC​TCC​AGT​CT-3′
	Reverse, 5′-GAT​GGC​AAA​CTT​TTG​ATC​ATT​GTT​A-3′
skn-1	Forward, 5′-GTT​CCC​AAC​ATC​CAA​CTA​CG-3′
	Reverse, 5′-TGG​AGT​CTG​ACC​AGT​GGA​TT-3′
GAPDH	Forward, 5′-TTC​TCG​TGG​TTG​ACT​CCG​AC-3′
	Reverse, 5′-AGG​GAG​GAG​CCA​AGA​AGG​TAA​C-3′

### 2.13 GFP-Reporter Transgenic Strains for Daf-16, Skn-1, hsp16.2, and Sod-3

Strains TJ356, LD1, CF1553, and TJ375 were used to further explore the molecular mechanisms of the ORR. The TJ356 and LD1 strains were used to respectively examine the mRNA expression and nuclei localization of daf-16 and skn-1 in the living nematodes. Synchronized TJ356 and LD1 worms were exposed to ODRR or OPRR for 4 days, and then the effect of oxidative stress by 200 μM juglone for 2 h was adopted. Next, the worms were washed with S-basal and mounted on the agar pads with 10 mM sodium azide. Fluorescence images were taken at X10 magnification with a fluorescence microscope. The nuclei localization of daf-16 and skn-1 was expressed as the number of fluorescence points in each nematode. A minimum of 20 worms per group was used in each experiment.

*C. elegans* strains CF1553 (sod-3::GFP) and TJ375 (hsp-16.2::GFP) were used to study the effect of ORR on sod-3 and hsp16.2. For CF1553, synchronized eggs were placed on plates containing ODRR or OPRR to the second day of adulthood. Then, the nematodes were transferred to new NGM plates containing 200 mM juglone, exposed for 6 h, and the fluorescence was measured. The pretreated transgenic nematode TJ375 was exposed to ODRR or OPRR and cultured for 2 days to the L4 stage at 20°C. Next, in order to induce heat shock response, the L4 nematodes were transferred to 20°C after being cultured at 35°C for 2 h. The fluorescent expression of each nematode was measured on the second day of adulthood. The treated strains CF1553 and TJ375 were mounted in a drop of 10 mM sodium azide. Then, the intensity of fluorescence of nematodes was observed using a fluorescence microscope system and analyzed using ImageJ software. Approximately 15 nematodes were measured in each group three times in parallel.

### 2.14 Statistical Analysis

The data were expressed as the mean ± SEM, and the standard error of the mean was shown by a bar in the figures. Statistical analyses of lifespan, paralysis, and stress resistance assay were carried out using the log-rank test. Other data were analyzed using one-way analysis of variance (ANOVA) followed by Bonferroni through SPSS 18.0 software (SPSS, Inc. Chicago, IL, United States). Significance was considered for *p* < 0.05.

## 3 Results

### 3.1 Characterization of Chemical Profile of ODRR and OPRR

**FIGURE 1:**
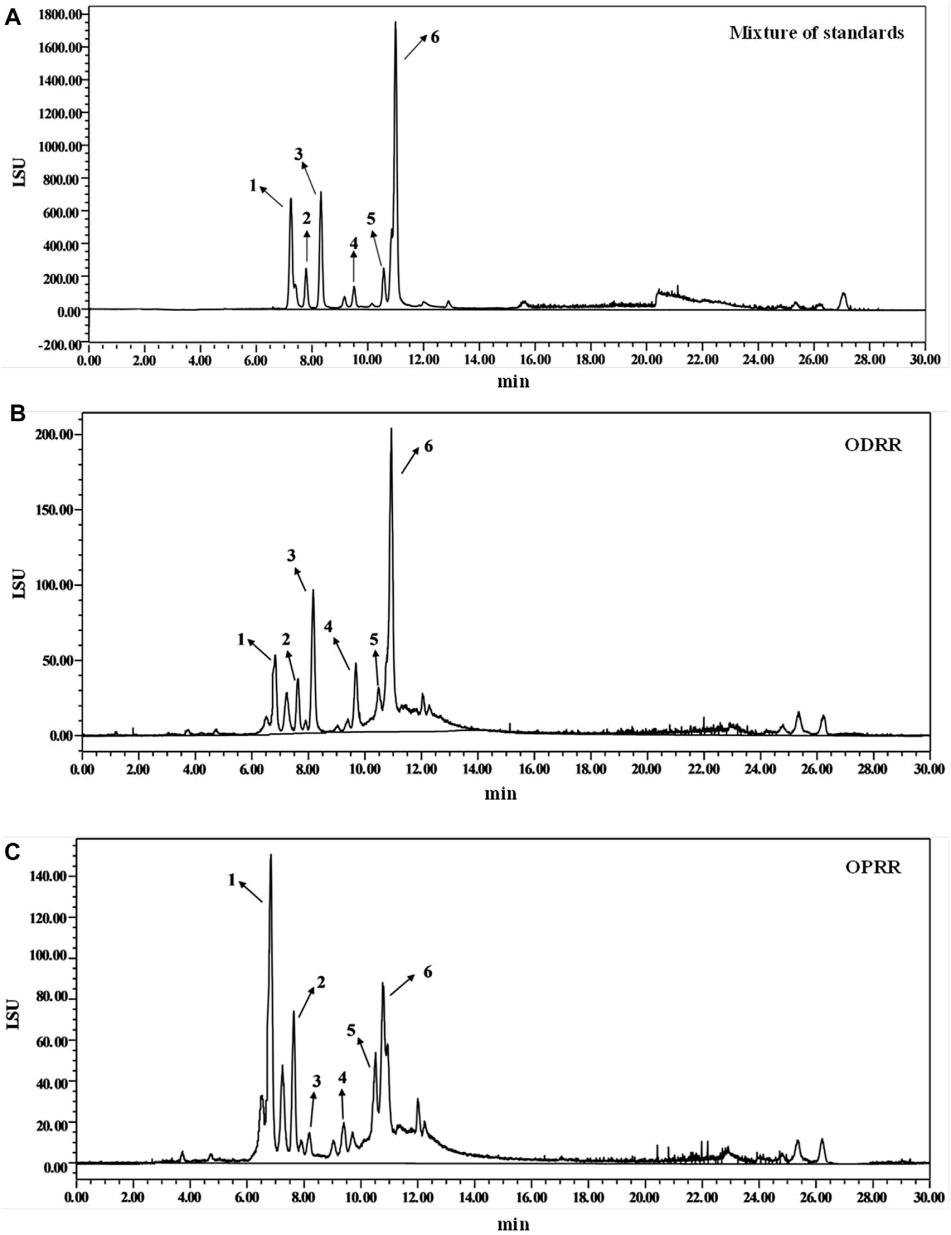
HPLC chromatograms of oligosaccharides from partially purified DRR and PRR. **(A)** Standards—1: raffinose, 2: glucose, 3: sucrose, 4: melibiose, 5: mannotriose, 6: stachyose. **(B)** ODRR. **(C)** OPRR.

The purified oligosaccharides extracted from DRR and PRR accounted for 24.30% and 28.83% of the original slices, respectively. The content and composition of oligosaccharides in RR changed after processing (Zhou et al., 2016). The difference in ingredients is the material basis for different efficacy. We determined the content of stachyose, raffinose, mannotriose, melibiose, sucrose, and glucose in ODRR and OPRR using HPLC-ELSD. The amount of stachyose and sucrose decreased, while the contents of raffinose, glucose, melibiose, and mannotriose increased (Figure 1). The total content of five kinds of oligosaccharide monomers, including stachyose, decreased after RR was processed (Table 2). The results showed that the content of each oligosaccharide in RR was changed during the steaming process.

**TABLE 2:** Quantitative phytochemicals in ODRR and OPRR with HPLC (mean ± SEM).

%	Raffinose	Glucose	Sucrose	Melibiose	Mannotriose	Stachyose	Total
ODRR	6.00 ± 0.78	6.78 ± 0.67	8.01 ± 0.96	5.69 ± 0.25	15.40 ± 1.51	29.11 ± 2.46	71.00 ± 6.18
OPRR	12.10 ± 0.60	10.01 ± 0.05	5.31 ± 0.01	7.14 ± 0.02	24.66 ± 0.32	21.99 ± 0.06	81.22 ± 3.89

### 3.2 ORR Significantly Leads to a Decrease in Paralysis

**FIGURE 2:**
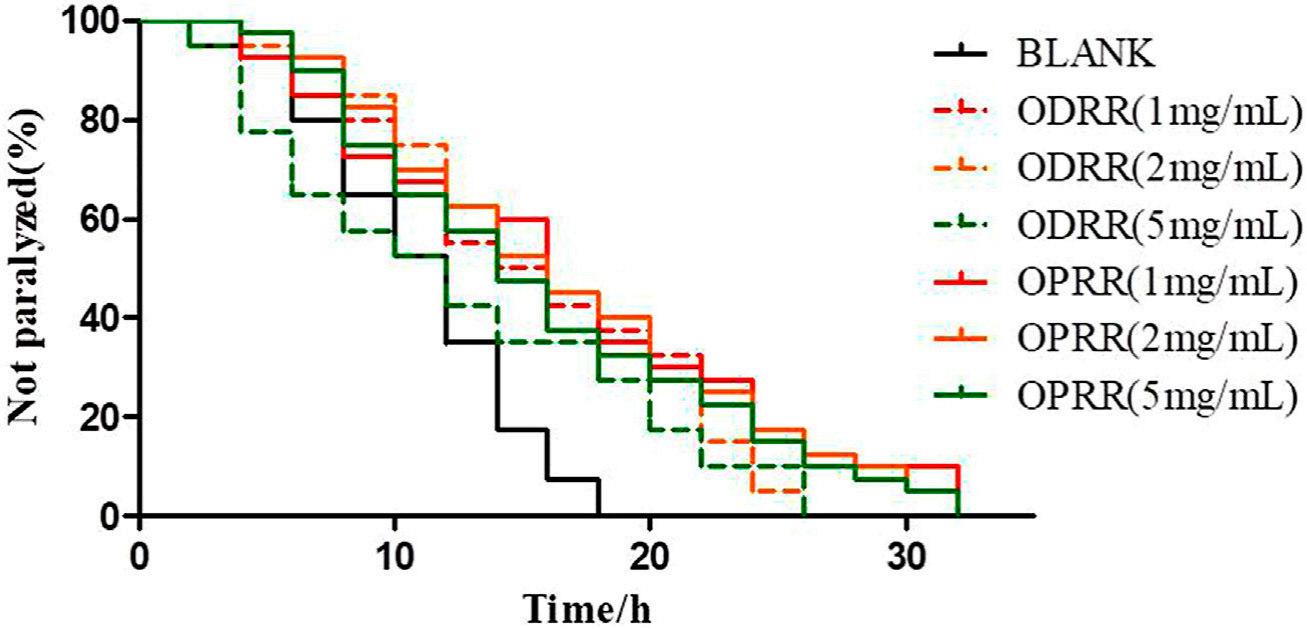
Paralysis rate of nematode CL4176. Synchronized CL4176 worms were treated with 1, 2, or 5 mg/ml ODRR and OPRR, respectively. After 48 h of culture at 16°C, the nematodes were then incubated at 23°C for 36 h to initiate the amyloid-induced paralysis. Each experiment was representative of three independent trials.

*C. elegans* strain CL4176 expressing Aβ_1-42_ in muscle cells was selected to evaluate whether ORR has a protective effect on Aβ-induced toxicity. Herein, we found that ODRR and OPRR significantly alleviated paralysis of nematodes compared with the control group, but neither of them was concentration-dependent. The results showed that ORR significantly alleviated Aβ-induced paralysis in CL4176 (*p* < 0.001). Although the paralysis percent of CL4176 treated with 2 mg/ml was lower than that with 1 mg/ml in the early stage of the assay, ODRR and OPRR were not concentration-dependent in the survival time. Considering the cost, we selected 2 rather than 5 mg/ml as the concentration for the following experiments (Figure 2).

### 3.3 ORR Increases Lifespan and Stress Resistance

**FIGURE 3:**
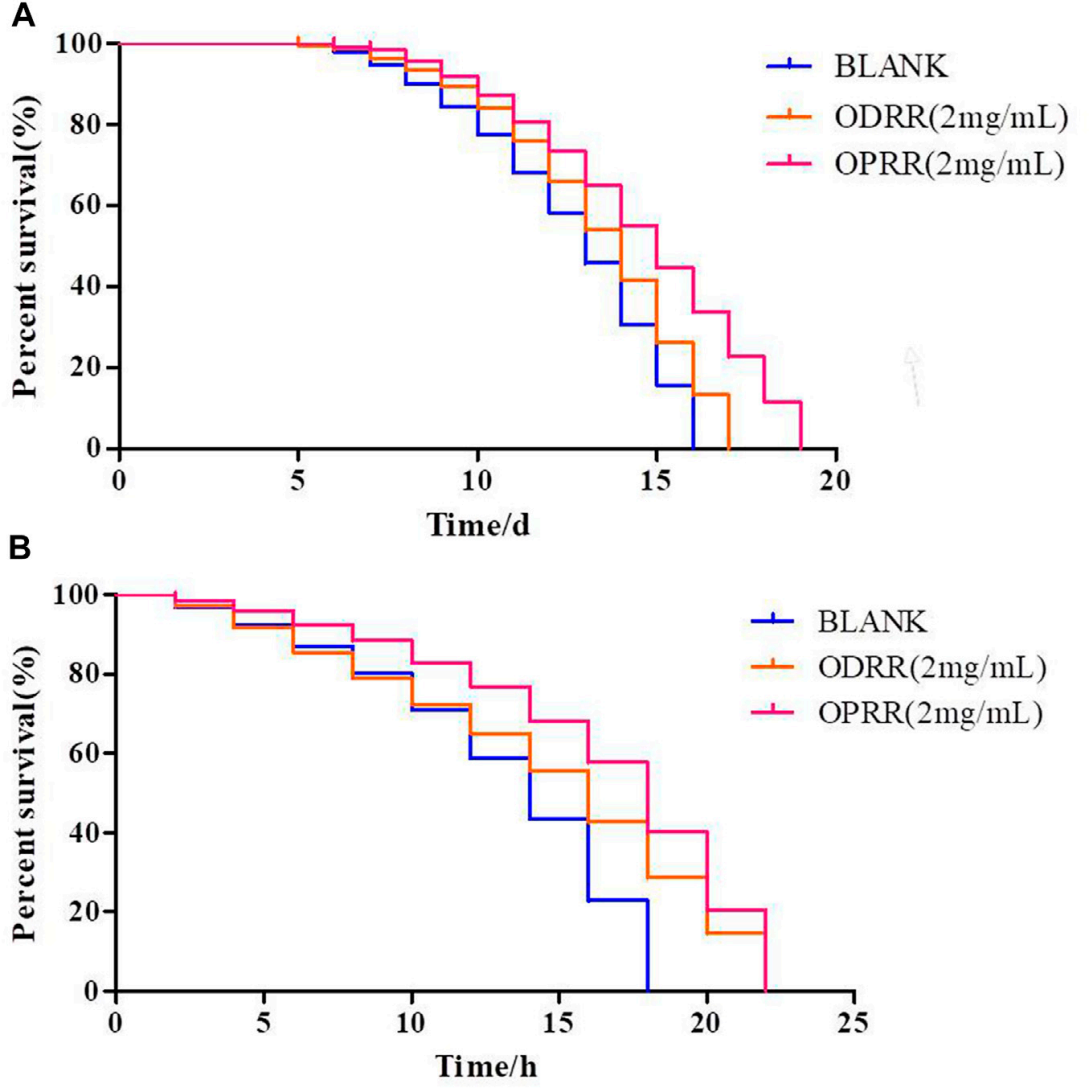
Lifespan and stress resistance of transgenic *C. elegans* CL4176 were increased under the treatment of ORR. **(A)** Survival curve of CL4176. **(B)** Survival curve under oxidative stress. The survival rate curves of nematodes were significantly different through the log-rank test. Each experiment is representative of three independent trials.

The development of neurodegenerative diseases is related to aging (Link, 2006). Therefore, we explored whether the effects of ODRR and OPRR on AD models were actually due to general anti-aging effects. We first measured the effect of ODRR and OPRR on the lifespan of CL4176. The lifespan of worms exposed to ODRR and OPRR significantly increased compared with the control. The lifespan of nematodes was extended from 16 to 17 days after ODRR fed and was extended to 19 days after OPRR fed (Figure 3A). The survival rate curves of nematodes were significantly different through the log-rank test (*p* < 0.001).

Increased longevity is associated with increased resistance to various forms of environmental stressors (Zhou et al., 2011). Oxidative stress is related to AD and the aggregation of Aβ, meaning that oxidative stress affects the accumulation of Aβ, while Aβ may also induce oxidative stress (Ganguly et al., 2017). Juglone has strong oxidability, causing oxidative damage to *E. coli* (Li and Zhang, 2013). This study determined whether ORR could extend the survival time of nematodes in the oxidative environment caused by juglone. The mean survival time for nematodes cultured on *E. coli* OP50 under oxidative stress conditions was 18 h, which was extended to 22 h after exposure to ODRR and OPRR (Figure 3B). Therefore, we believe that the potential of ORR for delaying paralysis and increasing lifespan is likely attributed to antioxidant activity. The survival rate curves of nematodes under oxidative stress and thermal stress were significantly different through the log-rank test (*p* < 0.001).

### 3.4 ORR Has No Effect on the Growth and Development of *C. elegans*

Locomotor behavior, reproductive output (Flatt, 2011), and food intake (Piper and Bartke, 2008) are closely related to aging. The sinusoidal movement frequency of N2, a wild-type nematode, decreased with the increase in survival time. The body bending frequencies of the adult worms on the fourth and the eighth days were determined. There was no significant difference in the movement frequency of nematodes treated with ODRR and OPRR on the fourth day compared with OP50, while the body bending frequency of nematodes treated with OPRR increased significantly on the eighth day (Supplementary Figures S1A,B). The results exhibited the effect of ORR on delaying senescence.

The number of nematodes spawning can be used to monitor neuronal dysfunction. It was reported that reduced fertility and slower growth could extend the lifespan of *C. elegans* (Mair and Dillin, 2008). The total number of offspring was measured to assess whether the treatment with ORR could interfere with fertility. The number of spawning increased after ORR treatment compared with OP50, but there was no significant difference, suggesting that ORR did not affect the reproductive function (Supplementary Figure S1C).

Food intake affects the lifespan of nematodes, and pharyngeal twitch can be used to assess the food intake of nematodes. Dietary restrictions (DR) could reduce available nutrients, regulate metabolism, and extend the lifespan of different organisms, including yeast and mammals. We determined the effect of ORR on the number of throat pumps in wild-type nematodes. No change in pharyngeal pumping rate was observed after treatment with ORR, indicating that the effects of ORR were not based on caloric restriction (Supplementary Figure S1D).

### 3.5 The Protective Effects of ORR Are Independent of the Uptake of *E. coli* OP50 by Nematodes

It has been known that the source and the amount of food consumed by nematodes affect the lifespan, protein aggregation phenotype, and toxicity (Reinke et al., 2010; Kim, 2013). We analyzed the effect of ORR on the growth of *E. coli* OP50. Compared with the control, there was no significant difference in OD_600_ when ODRR and OPRR were added to the *E. coli* OP50 culture medium within 5 days compared with the control group (Supplementary Figure S2), suggesting that the amount of food feed *C. elegans* has not been altered by ORR.

### 3.6 ORR Inhibits Aβ-Induced Increase in ROS Production

**FIGURE 4:**
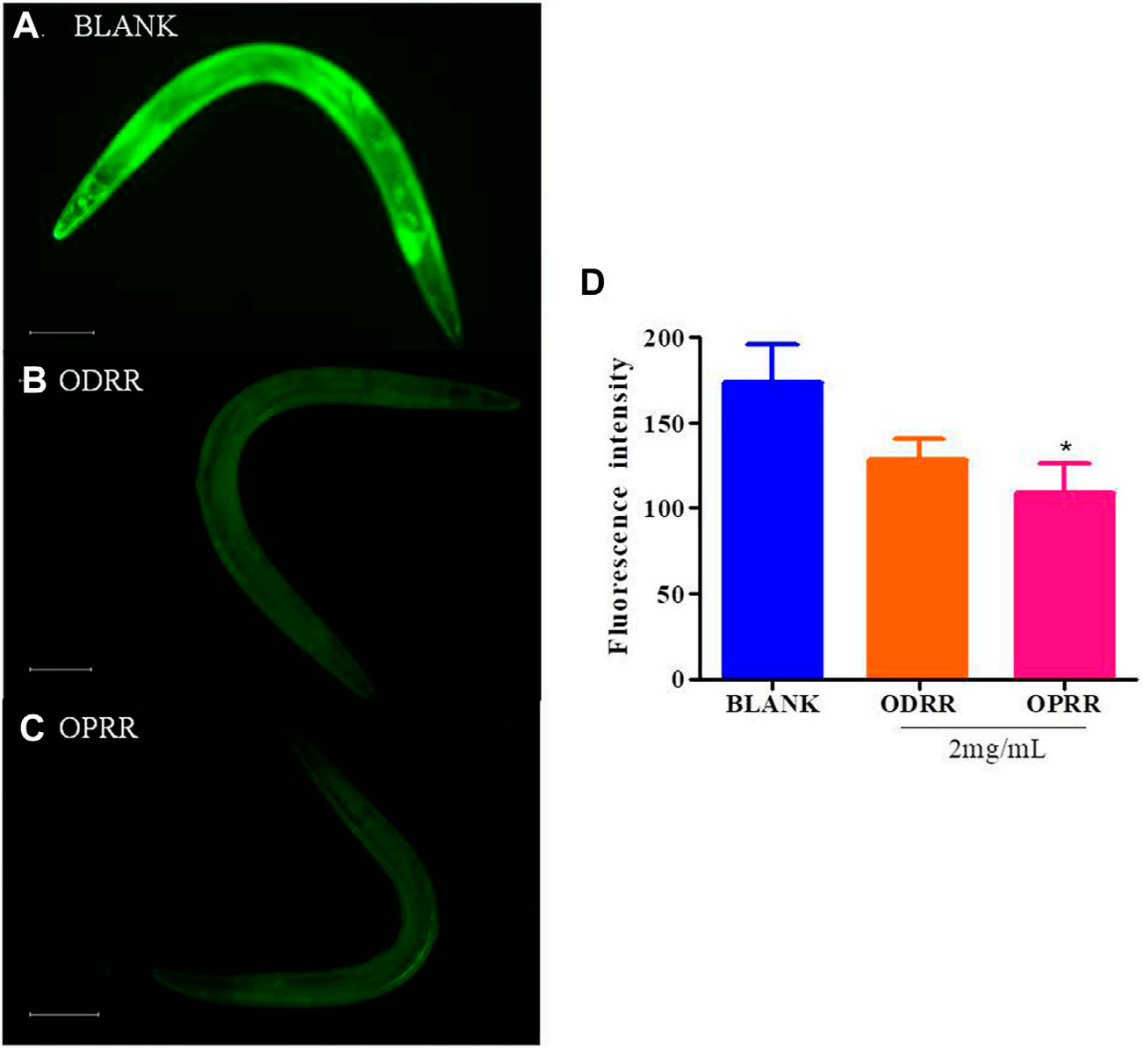
ORR decreases ROS in *C. elegans*. Synchronized CL4176 were cultured at 16°C for 48 h and then transferred to 23°C for 36 h, treating with or without ORR. Endogenous ROS was analyzed using H2DCF-DA. Representative images of ROS analyzed with H2DCF-DA in *C. elegans* treated with OP50 **(A)**, ODRR **(B),** or OPRR**(C)**. Scale bars: 100 µm. The fluorescence intensity per nematode was measured **(D)**. Data are presented as mean ± SEM. *p < 0.05 represents comparison with the blank group. Each experiment was representative of three independent trials.

Accumulation of extremely high ROS levels could lead to oxidative stress, which can inactivate the proteasome system, leading to the aggregation of toxic proteins (Sen and Packer, 1996; Birla et al., 2020). On the contrary, dysfunctional mitochondria caused by protein toxicity may produce excessive ROS (Devi et al., 2006). We have demonstrated that ORR has an antioxidative stress effect. Therefore, in order to further analyze whether ORR can reduce ROS in nematodes, we measured the ROS activity using transgenic strain CL4176 treated with ORR and the control. The ROS activity in CL4176 was decreased after ORR treatment compared with the control, but only OPRR significantly reduced ROS (*p < 0.05*). ODRR and OPRR suppressed ROS by about 26% and 37%, respectively. These results suggested that the potential of ORR on paralysis decrease and antioxidant activity in *C. elegans* could be attributed to the reduction of ROS levels (Figure 4).

### 3.7 ORR Decreases Amyloid Deposition in Transgenic *C. elegans*

**FIGURE 5:**
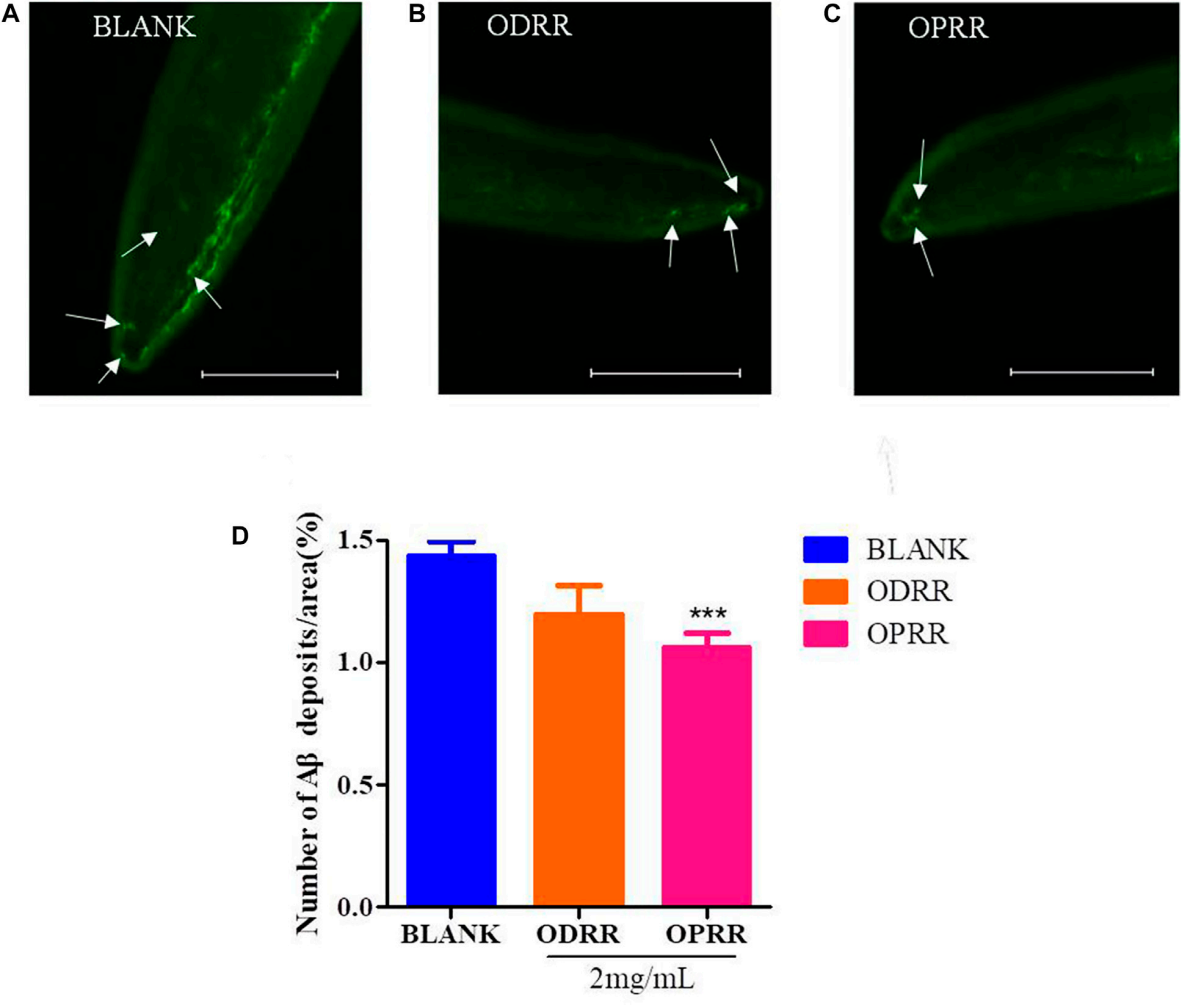
ORR decreases amyloid deposition in CL2006. CL2006 were synchronized and treated with ODRR and OPRR at 16°C to L4, respectively, and then the temperature was raised to 20°C for 24 h. Amyloid deposits with ThS specific staining were measured under a fluorescence microscope. Representative images of *C. elegans* with ThS staining in CL2006 fed with OP50 **(A)**, ODRR **(B),** or OPRR **(C)**. Scale bars: 100 µm. The number of deposits (arrows) was scored in the worm head **(D)**. All values are presented as mean ± SEM, ***p < 0.001, which represents a comparison with the blank group. Each experiment was representative of three independent trials.

The transgenic nematode CL2006 expresses high levels of human-derived Aβ in the body wall muscle cells, which results in excessive deposition of Aβ protein in the muscle cells, further leading to paralysis (Reiss et al., 2018). We measured the expression of Aβ in nematode cells by ThS staining to explore whether delayed paralysis by ORR in nematodes was related to the decrease in Aβ protein expression. The fluorescent spots on the head of CL2006 treated with ORR decreased compared with the control group, but there was a significant difference (*p < 0.0001*) only with OPRR treatment (Figure 5). These results indicated that ORR could reduce Aβ protein aggregation, which meant the reduction of Aβ-induced paralysis and the decrease of chemotaxis by ORR might be related to the decrease in Aβ aggregation.

### 3.8 ORR Suppresses the Defect of Chemotactic Behavior Induced by Neuron Aβ Expression

**FIGURE 6:**
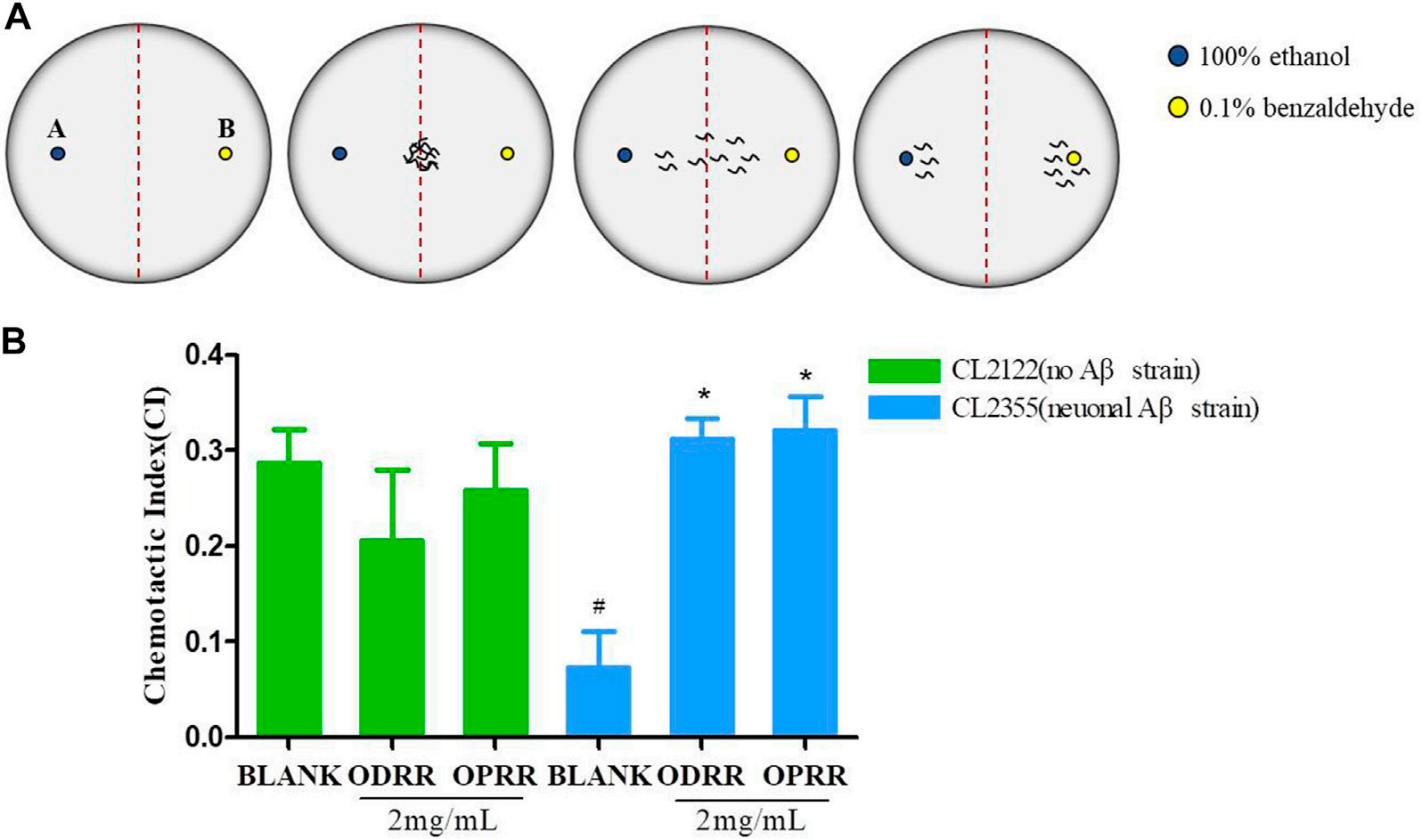
ORR improves the defect of chemotaxis behavior in AD transgenic nematodes. **(A)** Plates were divided into two quadrants. 1 μL 100% ethanol with 1 µl of 0.25 M sodium azide and 0.1% benzaldehyde with sodium azide were dropped in 1.5 cm from the center, respectively. The nematodes were transferred to the center of the medium. **(B)** Chemotaxis was analyzed with the formula CI = (number of nematodes in benzaldehyde position – number of nematodes in ethanol position)/total number of nematodes after 1 h. All values are presented as mean ± SEM, #p < 0.05, represents comparison with CL2122 blank group; *p < 0.05, represents comparison with CL2355 blank group. Each experiment was representative of three independent trials.

It has known that the oligomerization of Aβ peptides is toxic to neurons. *C. elegans* has sensory organs, and its smell-sensory neurons are mediated by activating several interneurons and sensory neurons to stimulate motor neurons (Tsalik and Hobert, 2003). We next investigated whether ORR had the ability to recover the learning and memory impairment induced by the Aβ peptide. The chemotaxis of CL2355, expressing Aβ in the neuronal cells, was significantly reduced compared with the control nematode CL2122, which did not express Aβ. The chemotaxis of transgenic strain CL2122 was not improved after being treated with ORR. However, the reduction in chemotaxis of CL2355 caused by Aβ expression was significantly improved after ORR treatment compared with the control (*p < 0.05*) (Figure 6B). The results indicated that ORR could protect nerves from Aβ-induced toxicity and decrease the chemotaxis defects caused by Aβ expression.

### 3.9 OPRR Alleviates Serotonin Hypersensitivity Caused by Aβ Expression

**FIGURE 7:**
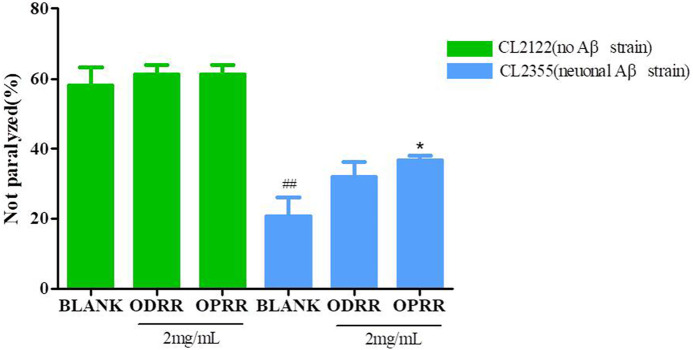
ORR improves serotonin-induced paralysis. Synchronized CL2355 and CL2122 treated with or without ODRR or OPRR at 16°C for 48 h were transferred to 23°C for 36 h. The paralysis number was analyzed after collecting the worms to a NGM medium containing 5 mg/ml 5-HT for 5 min. All values are presented as mean ± SEM; ##*p* < 0.01 represents comparison with CL2122 blank group; *p < 0.05 represents comparison with CL2355 blank group. Each experiment was representative of three independent trials.

The wild-type nematode N2 is sensitive to serotonin (5-HT), an important neurotransmitter that plays an important role in regulating nematode behaviors of olfactory learning, egg-laying, locomotion, and mating (Zhi et al., 2017). Exogenous 5-HT could inhibit *C. elegans* locomotion, leading active worms into paralysis. The overexpression of Aβ in neurons could enhance the sensitivity of CL2355 to exogenous serotonin and promote the paralytic phenotype (Wu et al., 2006). Then, we measured the paralysis of ORR on CL2355 induced by serotonin to confirm whether ORR could improve neuronal dysfunction caused by the Aβ protein. The number of non-paralytic CL2355 was only 20.67%, whereas CL2122 was 58.00% after 5 min in 5 mg/ml 5-HT (Figure 7). The number of paralytic worms decreased after ORR treatment; however, OPRR alone had a significant effect compared with the control (*p* < 0.05), which indicated that OPRR effectively relieved serotonin sensitivity caused by Aβ protein.

### 3.10 Gene Expression Analysis in *C. elegans* Treated With ORR

**FIGURE 8:**
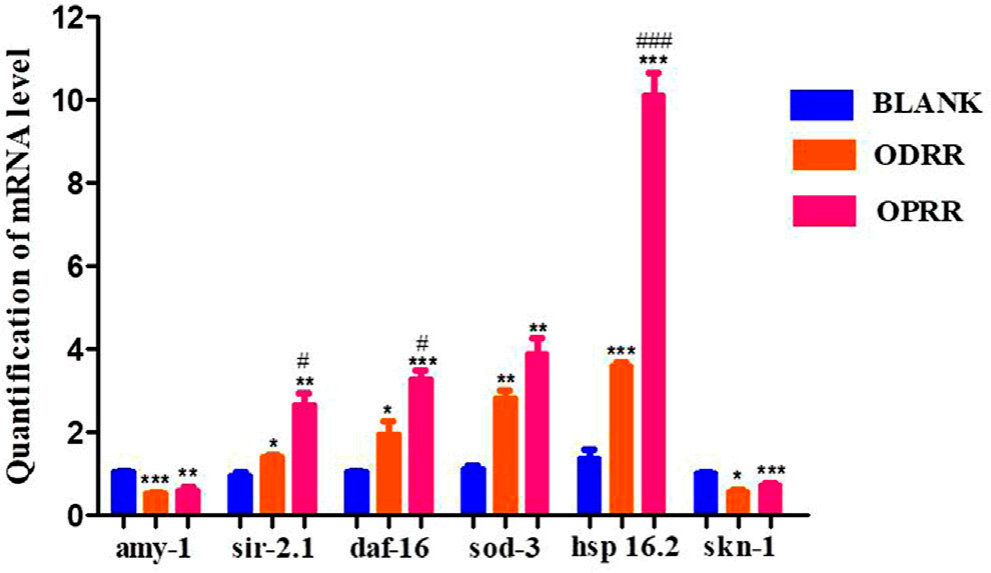
ORR reduces the expression of amy-1 and skn-1 and promotes the expression of sir-2.1, daf-16 sod-3, and hsp-16.2. The synchronized CL4176 were incubated with or without ORR at 16°C for 48 h and then incubated at 23°C for 36 h. The expression of six genes, including amy-1, sir-2.1, daf-16, sod-3, hsp-16.2, and skn-1, was analyzed using RT-PCR technology. All values are presented as mean ± SEM. *p < 0.05, **p < 0.01, ***p < 0.001 represent comparison with the blank group. #*p* < 0.001, ###*p* < 0.001 represent comparison with ODRR. Each experiment was representative of three independent trials.

To investigate the factors directly contributing to the induction of ORR to Aβ aggregation reduction, oxidative stress resistance, and ROS decrease, transgenic *C. elegans* with constitutive muscle-specific Aβ expression were used to analyze the effect of ORR on the expression of six genes, including amy-1, sir-2.1, daf-16, sod-3, hsp-16.2, and skn-1. After treatment with ORR, the expression of amy-1 decreased significantly, indicating that the expression of the Aβ gene was reduced. It was consistent with the results of ThS staining of Aβ protein in nematodes. After treatment with ORR, the expressions of sir-2.1, daf-16, sod-3, and hsp16.2 increased, and the expression of skn-1 decreased (*p* < 0.05, *p* < 0.01, *p* < 0.001) (Figure 8). The above experimental results indicated that the mechanism of ORR delaying the paralysis and prolonging the life of nematodes could be related to the inhibition of protein aggregation and antioxidation.

### 3.11 ORR Increases Daf-16:: GFP, Sod-3::GFP, and Hsp-16.2::GFP Expressions

**FIGURE 9:**
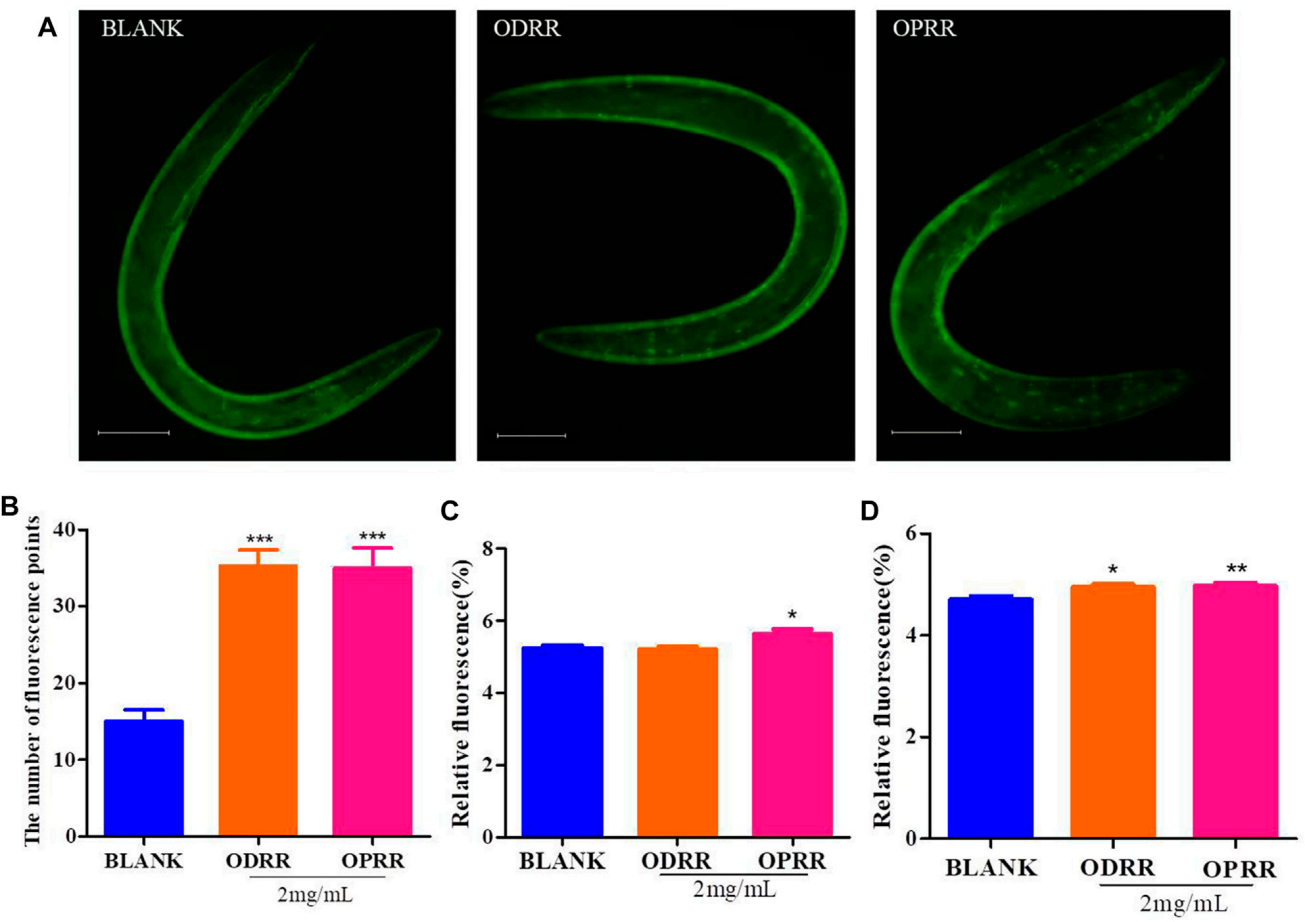
ORR accelerates daf-16:: GFP, sod-3::GFP, and hsp-16.2::GFP expressions. Nuclei localization of daf-16 in strain TJ356 was measured. The synchronized TJ356 were treated with ODRR or OPRR to the second day of adulthood, and then the effect of oxidative stress by 200 μM juglone for 2 h was adopted. Fluorescence images were obtained using a fluorescence microscope **(A)**. The number of fluorescence points in each nematode represents the nuclei localization of daf-16 **(B)**. CF1553 (sod-3::GFP) were synchronized to the L4 stage treated with or without ORR and then transferred to the NGM medium containing 200 mM juglone for 6 h. The temperature was raised to 35°C for 2 h after TJ375 (hsp16.2::GFP) was synchronized to L4 at 20°C and then restored to 20°C until the second day for adults. The fluorescent expression of sod-3 **(C)** and hsp16.2 **(D)** in each nematode was measured, respectively. Scale bars: 100 µm. All values are presented as mean ± SEM. *p < 0.05, **p < 0.01, and ***p < 0.001 represent comparison with blank group. Each experiment was representative of three independent trials.

It was reported that daf-16, skn-1, the important transcription factors, play pivotal roles in regulating longevity and ameliorating Aβ aggregation and toxicity (Zamberlan et al., 2020). Daf-16 could promote the expression of skn-1 (Tullet et al., 2017). Upon activation, daf-16 and skn-1 transfer from the cytoplasm to the nucleus and activate the expression of downstream genes (Zhao et al., 2017). In this study, we measured the nuclear localization of daf-16 and skn-1 using CF1553 and LD1, respectively. The results indicated that the treatment with ORR accelerated the daf-16 nuclear translocation (*p* < 0.0001) (Figures 9A,B). However, similar results did not appear in LD1, which was consistent with the analysis results of RT-PCR. Therefore, we speculated that the effect of ORR on suppressing Aβ toxicity and enhancing resistance to oxidative stress was partly due to the activation of daf-16.

In order to clarify the molecular response related to the protection of ORR to *C. elegans*, we measured the expression of two genes, including sod-3 and hsp-16.2, related to longevity and inhibition of protein aggregation. Sod-3 participates in protecting *C. elegans* from oxidative stress and suppressing the Aβ toxicity (Minniti et al., 2015). As a chaperon, hsp-16.2 could be induced by the abnormal Aβ proteins to increase their sequestration, degradation, and refolding (Fonte et al., 2008). Only OPRR significantly increased the fluorescence in CF1553 expressing sod-3::GFP (*p* < 0.05) (Figure 9C). The fluorescence in TJ375 expressing hsp-16.2::GFP (*p* < 0.05, *p* < 0.01) (Figure 9D) significantly increased after both ODRR and OPRR treatment, indicating that ORR could delay nematodes’ paralysis and prolong lifespan by activating sod-3 and hsp-16.2.

## 4 Discussion

Alzheimer’s disease (AD) is a chronic, age-related neurodegenerative brain disorder characterized by dementia, progressive loss of memory, and oxidative stress (Mir et al., 2021). Currently, there are approximately 50 million patients with AD worldwide, and this number is projected to double every 5 years, increasing to 152 million by 2050. The burden of AD affects patients themselves, their families, and the social economy, costing an estimated US$1 trillion annually in the world (Livingston et al., 2020; Yiannopoulou and Papageorgiou, 2020). The neuropathology features of AD are senile plaques, neurofibrillary tangles, synaptic loss, and others. At present, the hypotheses of AD pathogenesis include the cholinergic and amyloid hypotheses (Breijyeh and Karaman, 2020). We have known that the earliest stage of AD (the cellular phase) happens concurrently with the accumulation of Aβ, which induces the spread of tau pathology (Scheltens et al., 2021). Extraneuronal amyloid plaques, aggregates of Aβ, are cleavage products of amyloid precursor protein (APP) (Serpell et al., 2000). Although AD is a public health issue, only two kinds of drugs are approved to treat AD, including inhibitors to cholinesterase enzyme and antagonists to N-methyl d-aspartate (NMDA). The available treatments just improve the symptoms of AD, but there is no cure for it (Breijyeh and Karaman, 2020). In addition, BACE1, against Aβ accumulation, has been so far the primary therapeutic target for AD therapies. However, the phase II/III trials of all potential drugs developed have not been successful (Srivastava et al., 2021). GV-971 (sodium oligomannate), an Aβ aggregation inhibitor, could improve cognitive function in mild-to-moderate AD. It has received conditional marketing approval in China. For a long time, *in vitro* and *in vivo* studies have verified that natural compounds possess therapeutic potential for AD. For instance, Trehalose, a naturally existing disaccharide, could decrease Aβ and plaque formation in mouse brains (Liu et al., 2020). Verbascoside, an active phenylethanoid glycoside derived from *Verbascum sinuatum*, has been evaluated to improve the viability of Aβ_1-42_-damaged U251 cell and reduce the deposition of Aβ in the brains of APP/PS1 mice (Wang et al., 2020). Moreover, there are many other natural compounds used in folk medicine (traditional Chinese medicine (TCM)) that are effective in treating AD (Ma et al., 2019).

A whole series of animal models were generated to study both the mechanisms of AD and the effects of different treatments, including rats, mice, rabbits, dogs, and also non-human primates. Researchers have obtained a wealth of information using these models. Nevertheless, they have important limitations, such as a long study time, high cost, and ethical problems. Consequently, smaller model organisms (flies, zebrafish, and worms) have been developed. *C. elegans* was introduced as a model organism by Sydney Brenner in 1963 (Brenner, 1974). Compared with traditional animal models, this free-living nematode possesses many characteristics, such as a short lifespan, small size, genetic tractability, and more than 65% of the genes associated with human disease, including APP and presenilins (Shaye and Greenwald, 2011; Kim et al., 2018; Shen et al., 2018). Recently, genome-wide association studies (GWAS) have discovered that about 50 genes associated with an increased tendency to late-onset AD have orthologues in *C. elegans* (Sims et al., 2020). All these advantages make this nematode an ideal living system for AD studies. In addition, both tau phosphorylation and APP processing pathway are highly conserved in *Homo sapiens* and *C. elegans* (Apostolakou et al., 2021). Although *C. elegans* cannot generate endogenous Aβ because of the absence of β-secretase, exogenous expression methods have remedied this limitation by producing human β-amyloid peptides in muscle cells and pan neurons (Griffin et al., 2017). In recent years, researchers have used *C. elegans* to study the function of APP and presenilins and the function and mechanism of toxicity of Aβ and Tau (Alvarez et al., 2022). As of now, there is a good deal of toxicity models expressing normal or mutated Aβ in *C. elegans*. These models have made it possible to screen the potential natural compounds for reverse toxic effects, including nicotine, caffeic acid, n-butanol extract of *Hedyotis diffusa* Willd, erythraline, and erysodine, obtained from Fabaceae (DanQing et al., 2021; de Almeida et al., 2021; Li et al., 2021; Lu et al., 2021).

RR, a traditional Chinese medicine, is frequently used in traditional Chinese medicine prescriptions to delay aging and slow down dementia (Liu, 2011; Sanabria, 2013). Polysaccharides and catalpol in RR have been proven to be effective in anti-aging and AD treatment (Wang et al., 2018; Yuan et al., 2019). The researchers have also confirmed the neuroprotective effects of ORR (Yang et al., 2008). Herein, the transgenic nematode CL4176 that induces the expression of Aβ was employed as an AD model to study the protective effects of ODRR and OPRR against Aβ toxicity *in vivo*. They both delayed Aβ-induced paralysis, in which OPRR was more effective than ODRR. Although there was no concentration dependence, we did not attempt to study over a larger dose range because ODRR and OPRR were significantly different at less than 5 mg/ml. In addition, chemotactic experiments also confirmed that ORR improved the learning and memory ability of transgenic nematode CL2355, a transgenic nematode expressing Aβ in pan-neurons. Because aging plays a significant role in AD and retains the most primary risk factor for AD, we then analyzed the effects of ORR on the lifespan of nematodes. The results showed that the lifespan of nematodes was prolonge. All the results above exhibited that OPRR was more effective than ODRR, suggesting that the functional diversity of DRR and PRR could be related to the differences in oligosaccharides.

Oxidative stress is the key to the pathological development of AD. The accumulation of mitochondrial DNA associated with aging increases the production of ROS, which activates amyloidogenesis and produces Aβ peptides (Cai and Tammineni, 2017). In this context, ORR increased the survival of *C. elegans* under oxidative stress induced by juglone. Based on the antioxidant properties of ORR, the cumulative amount of ROS in the nematodes was measured. Expectedly, it significantly decreased the production of ROS. In addition, the expression of Aβ peptide is considered an important parameter for studying AD pathology (Paul et al., 2020). ORR not only delayed the paralysis of AD worms but also improved their ability to learn and remember. At the same time, it was confirmed by ThS staining that it reduced the deposition of Aβ in strain CL2006, a transgenic nematode expressing Aβ in the muscle cells of the body wall. The results above indicated that ORR plays a key role in antioxidants and reducing Aβ accumulation.

Then, we used RT-PCR technology to elucidate other putative mechanisms based on the positive effects of ORR on inhibiting Aβ peptide formation. Daf-16 is a downstream gene of the insulin-like signaling pathway (IIS) pathway related to lifespan extension. In *C. elegans*, daf-16 is also a central regulator in a gene network, including the skn-1 transcription factor, the ortholog of nuclear factor erythroid 2-related factor 2 (Nrf-2) protein in a mammal, which is related to longevity and oxidative stress responses (Tullet et al., 2017). The researchers have described that daf-16 could promote the expression of skn-1. The downstream target genes of daf-16 are sod-3 and hsp-16.2 (Zečić and Braeckman, 2020). Superoxide dismutase sod-3, targeting daf-16, has an important role in protecting against ROS (Wang et al., 2016). Small heat shock proteins (HSPs) could improve the survival of nematodes under stress and could be induced by Aβ expression (Webster et al., 2019). In addition, HSPs also prevent the accumulation of different types of toxic proteins such as Aβ and ployQ. The overexpression of hsp-16.2 could inhibit the toxicity of the Aβ peptide by assisting abnormal protein sequestration, degradation, and refolding in AD *C. elegans*. Furthermore, sir-2.1 acts as the active inductor of daf-16 and skn-1, resisting to oxidative stress, regulating longevity, and ameliorating Aβ toxicity (An and Blackwell, 2003; Cascella et al., 2014). In the present study, the results of RT-PCR analysis showed that sir-2.1, daf-16, sod-3, and hsp-16.2 mRNA levels increased, and the mRNA levels of amy-1 and skn-1 decreased in *C. elegans* CL4176 treated with 2 mg/ml of ORR compared with the control. ORR suppressed the transcription of amy-1 and upregulated hsp-16.2, indicating that the paralysis delay in CL4176 was at least partially due to amy-1 level reduction and hsp-16.2 up-regulation. We supposed that sir 2.1 activated daf-16 futher increased the transcription of downstream target gene sod-3. The above process might play a key role in arresting oxidative stress.

We next used strains TJ356 (daf-16::GFP), LD1 (skn-1::GFP), CF1553 (sod-3::GFP), and TJ375 (hsp-16.2::GFP) to deepen the molecular mechanisms of ORR. ORR only affected the cellular localization of daf-16, except for skn-1. The expression of sod-3 in the CF1553 strain treated with OPRR was increased treated OPRR, which indicated that OPRR needed daf-16 to increase sod-3 expression. We also observed that the fluorescence expression of transgenic nematode TJ375 was increased with ORR treatment, indicating that the upregulation of the hsp-16.2 gene expression by ORR also contributed to the protection of Aβ-induced toxicity in the AD nematodes.

## 5 Conclusion

In conclusion, Aβ-induced paralysis was alleviated in *C. elegans* by increasing the expression of daf-16 and hsp-16.2, lowering amy-1, and reducing ROS, all of which could interact with each other to participate in the protective mechanism against Aβ toxicity. In addition, the nuclear localization of daf-16, which promoted the expression of sod-3 and hsp-16.2, contributed greatly to suppressing the Aβ toxicity and oxidant stress of *C. elegans* treated with ORR. This study confirmed the role of ORR against toxicity through antioxidant activity and the inhibition of Aβ protein aggregation. The studies above provided a new perspective on the potential of ORR in the treatment of AD. We will further analyze the therapeutic effect of each monomer (stachyose, stachyose, raffinose, mannotriose, melibiose, sucrose, and glucose) on AD to determine whether the efficacy of oligosaccharides is related to integrality, which means the application of Chinese herbal in the treatment of diseases requires multiple components and targets rather than single component and single target. Moreover, we will consider the toxicity analysis of ORR in future experiments to analyze it comprehensively.

## Data Availability

The original contributions presented in the study are included in the article/Supplementary Materials. Further inquiries can be directed to the corresponding authors.

## Abbreviations

AD, Alzheimer’s disease; Aβ, β-amyloid protein; *C. elegans*, *Caenorhabditis elegans*; CI, Chemotactic index; DRR, Dried Rehmannia Radix; *E. coli* OP50, Uracil deficient *Escherichia coli* OP50; 5-HT, 5-hydroxytryptamine; LB, Luria-Bertani; NGM, nematode growth medium; ODRR, oligosaccharides in dried Rehmannia Radix; OPRR, oligosaccharides in prepared Rehmannia Radix; PRR, prepared Rehmannia Radix; ROS, reactive oxygen species; RR, Rehmannia Radix; ThS, Thioflavin S.
