# Supplementary material for: Neuroprotective Effects of Oligosaccharides in Rehmanniae Radix on Transgenic Caenorhabditis elegans Models for Alzheimer’s Disease
Source: Front Pharmacol. 2022 Jun 17;13:878631. doi: 10.3389/fphar.2022.878631 (PMC9247152; doi:10.3389/fphar.2022.878631)
Supplement: Supplementary file 1 [file DataSheet1.docx]

Fig S1. ORR has no effect on body bendings, brood size and pharyngeal pumping of *C.elegans*. Synchronized N2 was cultured on a medium with or without ODRR or OPRR, respectively, and transferred to M9 buffer to measure body bendings within 30 s on Day 4(A) or Day 8(B) of the adults (**p* < 0.05, represents comparison with blank group; ^###^*p* < 0.001, represents comparison with ODRR). (C) The number of brood size was analyzed by counting the total number of eggs laid on the first, the second, and the third day after adulthood. (D) Synchronized eggs were treated with ORR to the first day of adulthood, and the number of pharyngeal pumpings of the nematode within 20 s was measured. All values are presented as mean ± SEM, **p* < 0.05, represents comparison with blank group; ^###^*p* < 0.001, represents comparison with ODRR. Each experiment was representative of three independent trials.

Fig S2. ORR does not affect the growth of OP50. OP50 was incubated with ODRR and OPRR at 37 °C respectively, and OD_600_ was measured every 24 h. Data are presented as mean ± SEM. Each experiment was representative of three independent trials.
